# Percutaneous Magnetic Resonance Imaging-Guided Focal Laser Ablation (MRI-FLA) of Prostate Tumors: A Systematic Review and Network Meta-Analysis

**DOI:** 10.3390/jpm14121146

**Published:** 2024-12-10

**Authors:** Clément Marcelin, Clément Klein, Grégoire Robert, Franck Bladou, Nicolas Grenier, Eva Jambon

**Affiliations:** 1 Centre Hospitalier Universitaire de Bordeaux, Service de Radiologie et Imagerie Médicale de l’adulte, Place Amélie Raba Léon, 33076 Bordeaux, France; nicolas.grenier@chu-bordeaux.fr (N.G.); eva.jambon@chu-bordeaux.fr (E.J.); 2 Bordeaux Institute of Oncology, BRIC U1312, INSERM, Université de Bordeaux, 33000 Bordeaux, France; 3 Centre Hospitalier Universitaire de Bordeaux, Service d’Urologie Andrologie et Transplantation Rénale, Place Amélie Raba Léon, 33076 Bordeaux, France; clement.klein@chu-bordeaux.fr (C.K.); gregoire.robert@chu-bordeaux.fr (G.R.); franck.bladou@chu-bordeaux.fr (F.B.)

**Keywords:** prostate, tumor, MRI-guided, ablation, thermal ablation, and laser

## Abstract

**Background/Objectives:** MRI-guided focal laser ablation (MRI-FLA) is an emerging minimally invasive technique for treating localized prostate tumors, aiming to provide effective cancer control while minimizing side effects. This meta-analysis systematically evaluates the clinical outcomes, technical efficacy, and complication rates associated with MRI-FLA to better understand its therapeutic potential and safety profile in prostate cancer management. **Methods:** In July 2024, PubMed (MEDLINE) was searched for eligible trials using the PRISMA guidelines. The primary outcome was residual disease (RD). The secondary outcomes were technical efficacy, progression to metastatic disease, cancer-specific mortality, complications, and decreases in the prostate-specific antigen (PSA) level. **Results:** Nine clinical trials involving 296 patients with prostate tumors treated via MRI-FLA were analyzed. A random effects model showed that the overall RD prevalence after ablation was 20.37% (12.56–29.28%; *p* = 0.03) and the cancer-free survival rate was 75.62% (64.88–85.10%). The rate of major and minor adverse effects was 14.26% (0.61–37.3%, *p* < 0.01). **Conclusions:** MRI-FLA is safe, feasible, and effective, although further trials are required.

## 1. Introduction

Prostate cancer is one of the most frequently diagnosed cancers worldwide [1], and advances in screening, such as prostate MRI [2], have increased early detection rates, particularly in localized stages. Traditional whole-gland treatments, including surgery and radiotherapy, are effective but often lead to significant morbidities like urinary incontinence and erectile dysfunction. To address these issues, focal therapies such as MRI-guided focal laser ablation (MRI-FLA) have emerged as targeted alternatives. MRI-FLA allows precise treatment of the primary tumor, or “index lesion” [3], while sparing healthy tissue, facilitated by real-time anatomical imaging and thermometry via MRI. This approach not only aims to achieve effective tumor control but also prioritizes quality of life by reducing common side effects.

The American Urological Association and the American Society for Radiation Oncology guidelines for the treatment of localized prostate cancer (PCa) suggest that focal therapies (FTs) including high-intensity focused ultrasound (HIFU), radiofrequency ablation (RFA), microwave ablation (MWA), and laser treatments may be considered for patients with PCa of intermediate risk [4]. FTs selectively target identified areas of disease, preserving normal prostatic tissue and the surrounding structures including the delicate neurovascular bundles and urinary sphincter [5]. The aim is to maintain quality of life by reducing treatment-related side effects such as erectile dysfunction and stress urinary incontinence. Advances in multiparametric magnetic resonance imaging (mpMRI) have improved the precise localization of suspicious lesions based on the Prostate Imaging Reporting and Data System (PI-RADS), contributing to the growing feasibility of organ-sparing therapies [6,7,8,9,10,11]. Ultrasound (US)-guided HIFU has been widely studied [12,13,14]. However, mpMRI guidance enhances targeting and ensures better marginal accuracy in three dimensions. Compared to US-guided HIFU, the ablation volumes are smaller, there are fewer side effects, and the recovery is faster [6,7]. Additionally, MRI-guided FT hollowing exploits the real-time thermal feedback afforded by MR thermometry; the ablation power can be dynamically adjusted to optimize the ablation temperature [7]. The temperature standard deviation—reflecting thermal stability and precision—on prostate MR thermometry, is generally maintained below 1 °C [8].

Donaldson et al. [9] suggested a consensus FT terminology, where residual cancer is classified as a tumor > 3 mm in diameter with a Gleason score of 3 + 3, or a tumor with a Gleason score of 3 + 4 or 4 + 3 within the treatment zone. Post-treatment follow-up should include measurement of prostate-specific antigen (PSA), prostate MRI, and a prostate biopsy at 1 year [9,10]. Retreatment rates of up to 20% are considered acceptable; rates below 10% are clinically preferred.

Laser-based treatments take a transperineal, transrectal, or transgluteal approach with the patient under local anesthesia. These proceed in the outpatient setting with real-time temperature monitoring, but primarily address small lesions [11]. The first MRI-guided laser procedure was reported in 1998 [15]; it was possible to deliver energy to small regions and distinguish reversible and irreversible tissue changes. Later cadaveric studies [16] and case series [14] confirmed that temperature mapping revealed ablation zone devascularization. However, data on long-term outcomes remain limited, emphasizing the need for further studies on MRI-FLA’s sustained efficacy and safety.

In the absence of extensive long-term data on MRI-guided focal laser ablation (MRI-FLA) for prostate cancer, a systematic review approach can offer valuable insights. Conducted rigorously, this review can also highlight gaps in the current knowledge and establish priorities for future research. In this study, we present the findings of a systematic review aimed at assessing the clinical efficacy, safety, and outcomes of MRI-FLA in the treatment of localized prostate cancer. The results are intended to support a framework for standardizing MRI-FLA practices and inform recommendations that could guide further studies and clinical applications of this technology.

This review evaluates the current evidence of the long-term oncological efficacy of percutaneous MRI-focal laser ablation (FLA) for prostate tumor treatment.

## 2. Materials and Methods

This systematic review and meta-analysis of the literature describing the use of MRI-FLA to treat prostate tumors is based on the Preferred Reporting Items for Systematic Review and Meta-Analysis (PRISMA) guidelines [17]. Only peer-reviewed articles published in English up to August 2024 were reviewed. No publication date restriction was applied.

### 2.1. Eligibility Criteria

Article selection proceeded in three steps. First, PubMed was searched using various combinations of the following keywords: prostate, tumor, MRI-guided, ablation, TA, and laser. Duplicate articles were removed, and each abstract was assessed in terms of relevance. We excluded irrelevant reports and those lacking original cohort data. Finally, after a full-text assessment, we excluded a further two articles, including one with a follow-up of only 3 weeks. Salvage focal treatment was excluded.

**Figure 1:**
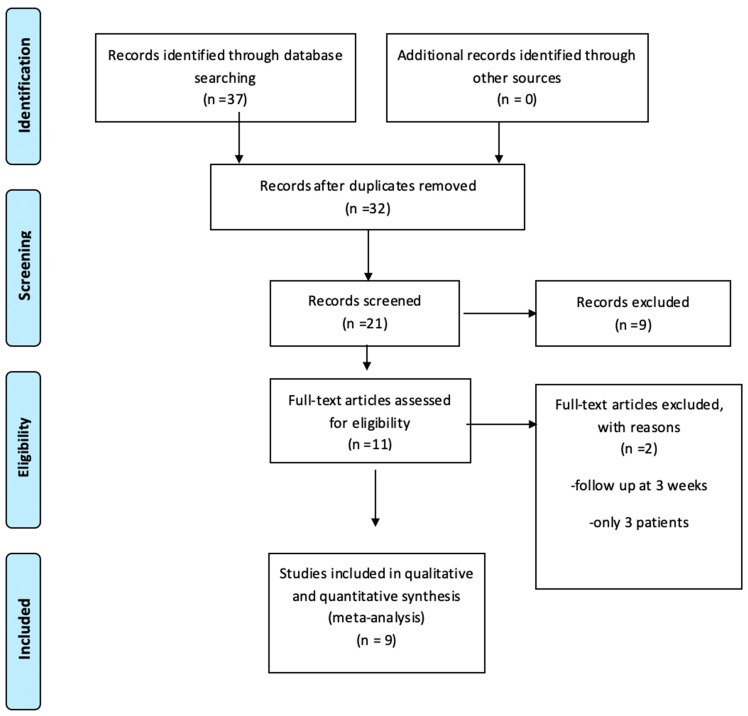
PRISMA flow diaphragm documenting the selection process for articles included in this meta-analysis using the following Keywords: prostate, tumor, MRI-guided, ablation, TA, and laser.

Figure 1 illustrates the PRISMA flow diagram outlining the article selection process for the meta-analysis. Initially, 37 records were identified through database searches using keywords such as “prostate”, “tumor”, “MRI-guided”, “ablation”, “thermal ablation (TA)”, and “laser”. After removing duplicates, 32 records remained for screening. From these, 21 records were evaluated, leading to the exclusion of nine based on relevance criteria. Eleven full-text articles were then assessed for eligibility, with two excluded due to limitations (i.e., follow-up period of only three weeks and sample size limited to three patients). Ultimately, nine studies were included in both qualitative and quantitative analyses for the meta-analysis.

### 2.2. Analysis

The main outcome measures were technical efficacy, residual disease (RD), local recurrence-free survival (LRFS), progression to metastatic disease, decrease in PSA level, complications, and the mean intervention time. Technical efficacy was defined as a successful creation of a non-enhancing focal defect in the ablated zone. RD was defined as a persistent, biopsy-confirmed prostate tumor in the ablation zone and recurrence as a new prostate tumor in the ablation zone. RD was evaluated at 3 and 6 months, and 1 and 3 years, in various studies. Cancer-specific survival was also assessed according to study protocol. All patients had biopsy-proven prostate tumors.

The inclusion criteria were tumor grade T1c or T2a, one or two lesions visible on mpMRI, Gleason score ≤ 3 + 4 = 7 (Grade 2), ≤3 cores positive in a standard 12-core biopsy or ≤4 cores positive on mpMRI image-guided biopsy (2 cores from each MRI-lesion), PSA < 15 ng/mL, and the absence of metastatic disease.

The exclusion criteria were more than two MRI-visible lesions, extracapsular extension or seminal vesicle invasion, metastatic disease, any condition that rendered MRI unsafe or infeasible, an acute urinary tract infection at the time of evaluation, severe lower urinary tract symptoms (International Prostate Symptom Score [IPSS] > 20), severely impaired renal function (estimated glomerular filtration rate [eGFR] ≤ 30 mL/min), uncontrolled coagulopathies, and/or any other serious illness or health condition that compromised the patient’s ability to participate in the study.

### 2.3. Statistical Analysis

The rates of RD, complications, and decrease in PSA were calculated using the “metaprop” package of R software (version 4.0.1). To deal with between-study heterogeneity, individual rates were subjected to double arcsin transformation, and random effects models were employed [18]. The random effects model was chosen as it assumes that the true effect size varies between studies rather than being fixed, providing a more conservative and generalized estimate of the pooled effect sizes. Heterogeneity was assessed using the I² statistic; outliers that significantly influenced the modeling were excluded. Publication bias was evaluated by drawing a funnel plot and performing the Egger regression test of asymmetry. Pooled effect sizes are reported with 95% confidence intervals (CIs), and statistical significance was set at *p* < 0.05.

We assessed the risk of bias for the included studies using the Cochrane Risk of Bias tool. This tool evaluates multiple domains, including selection bias, performance bias, detection bias, attrition bias, reporting bias, and other potential sources of bias.

## 3. Results

The risk of bias assessment indicated that all included studies were at high risk of bias. These findings highlight potential limitations in the internal validity of the data.

Our evaluation revealed that the majority of the included studies were at high risk of bias due to limitations in random sequence generation, allocation concealment, and incomplete blinding of participants, personnel, and outcome assessors. These factors may influence the reliability of the reported outcomes.

Inclusion criteria across the studies were T1c or T2a stage, PSA < 10 or <15 ng/mL, Gleason score < 8, and the presence of one or two lesions on MRI. An exception was noted in Walser et al. [19], which also included T1b grade cases. None of the patients had metastases, and most treatments were performed using a transperineal approach [20,21,22,23] while the remainder utilized a transrectal approach [19,24,25,26]. Only Al-Hakeem et al. [27] reported the use of both approaches.

Table 1 summarizes key characteristics and outcomes from studies included on MRI-FLA for prostate cancer. The studies included varied in the number of patients, with sample sizes ranging from 7 to 120 participants. The mean age of patients across studies generally ranged from 62 to 66 years, and tumor diameter varied from 11 to 15 mm. The average baseline prostate-specific antigen (PSA) levels before intervention ranged from 4.1 to 7.4 ng/mL, with follow-up periods ranging from 3 to 36 months. Cancer-free survival rates post-treatment were generally high, ranging from 34% to 96%, though residual disease and recurrence rates varied between 0% and 66%. Complications were also reported, with minor complications ranging from 0% to 67%, while major complications were typically low. Procedure times varied significantly, from 122 min to 292 min, reflecting differences in study protocols and patient characteristics. All studies evaluated the Visualase laser (Medtronic, Mineapolis, MN, USA).

### 3.1. RD

**Figure 2:**
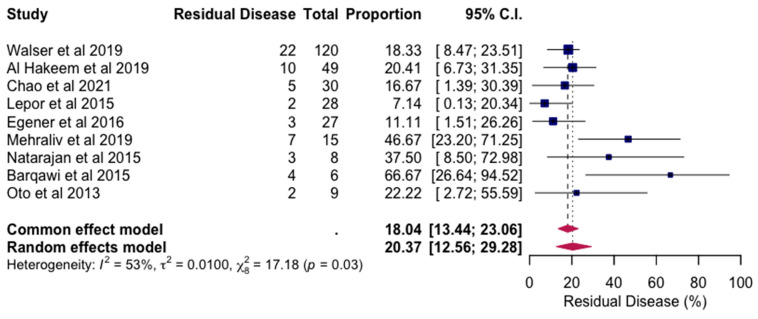
The random effects model for prevalence of residual disease following MRI-FLA [19,20,21,22,23,24,25,26,27].

Figure 2 presents a random effects model illustrating the prevalence of residual disease following MRI-FLA. This model aggregates data across studies, accounting for variations among them to estimate an overall prevalence rate. The random effects approach ensures that individual study differences are considered, providing a comprehensive view of the residual disease rate after MRI-FLA. This model supports the analysis by highlighting the consistency of MRI-FLA outcomes in minimizing residual disease across varied clinical trials. All reviewed articles reported RD status within the ablation zone after MRI-FLA.

When the data from the included studies were pooled, the analysis using a random effects model revealed an overall prevalence of residual disease (RD) of 20.37%, with a 95% confidence interval ranging from 12.56% to 29.28% (*p* = 0.03). This finding suggests that while MRI-FLA is effective in many cases, approximately one in five patients may still experience residual disease within the treated area.

### 3.2. Cancer-Free Survival

**Figure 3:**
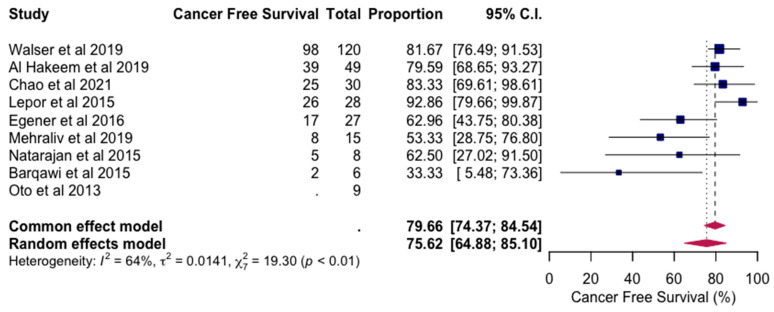
The random effects model for cancer-free survival following MRI-FLA [19,20,21,22,23,24,25,26,27].

Figure 3 displays a random effects model for cancer-free survival following MRI-guided focal laser ablation (MRI-FLA). This model synthesizes results from multiple studies, accounting for heterogeneity among them to provide an overall estimate of cancer-free survival rates post-treatment. By using a random effects model, the analysis accommodates variations across studies, presenting a consolidated view of MRI-FLA’s efficacy in sustaining cancer remission. This statistical approach enhances the robustness of the findings, demonstrating the consistency of cancer-free survival outcomes in diverse clinical contexts.

All articles, with the exception of the study by Oto et al. [21], reported on cancer-free survival status following MRI-FLA. The pooled cancer-free survival rate, derived from a random effects model, was calculated at 75.62%, with a 95% confidence interval ranging from 64.88% to 85.10% (*p* < 0.031). This outcome suggests that MRI-FLA is generally effective in achieving sustained cancer-free survival in a majority of patients.

### 3.3. Local Recurrence

Only the study by Chao et al. [26] provided data on local recurrence following MRI-FLA, reporting a recurrence rate of 40%, with 10 out of 25 patients experiencing local tumor recurrence. This relatively high rate highlights the potential for tumor persistence or regrowth within the treated area, suggesting that while MRI-FLA is generally effective, it may not completely eliminate the risk of recurrence in all cases. This finding underscores the need for close post-treatment monitoring and may prompt consideration of complementary strategies to further reduce recurrence rates.

### 3.4. Progression to Metastatic Disease

Progression to metastatic disease following MRI-guided focal laser ablation (MRI-FLA) was reported exclusively in the study by Chao et al. [26] where 2 out of 30 patients (6.7%) experienced metastasis post-treatment. This rate of progression highlights that, while MRI-FLA appears effective in controlling localized prostate cancer, there remains a risk of disease spread in a small subset of patients.

### 3.5. Technical Efficacy

Technical efficacy rates for MRI-FLA varied between studies, with Oto et al. [21] reporting a high efficacy rate of 88.9% (eight out of ten patients achieving successful ablation) and Walser et al. [19], reporting a somewhat lower rate of 63.3% (76 out of 120 patients). The high rate reported by Oto et al. suggests that MRI-FLA can achieve precise and effective ablation under optimal conditions, whereas the lower rate observed by Walser et al. may reflect challenges in maintaining consistent outcomes across a larger and possibly more diverse patient group.

### 3.6. Tolerance and Complications

**Figure 4:**
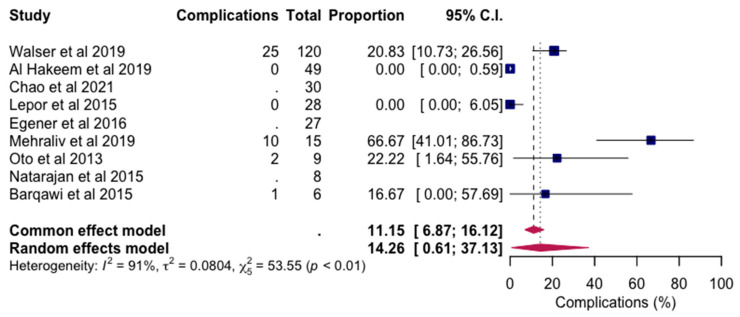
Random effects model for complication following MRI-FLA [19,20,21,22,23,24,25,26,27].

Figure 4 shows a random effects model analyzing complication rates following MRI-FLA. This model aggregates data from various studies, adjusting for inter-study differences to provide an overall estimate of complication incidence post-MRI-FLA. The random effects approach considers variability across studies, allowing for a comprehensive understanding of the safety profile associated with MRI-FLA. This analysis highlights the consistency of complication rates across clinical settings, reinforcing the reliability of MRI-FLA as a generally safe treatment for prostate tumors with manageable risks.

All studies included in this review reported the occurrence of adverse effects following MRI-FLA, underscoring the importance of assessing the safety profile of this procedure. Various classification systems were employed to categorize these effects: Al Hakeem et al. [27] used the Clavien-Dindo classification to grade surgical complications [28], while Walser et al. [19] adopted the Common Terminology Criteria for Adverse Events (CTCAE) for standardized reporting of side effects. Oto et al. [21] and Eggener et al. [23] applied the National Cancer Institute (NCI) Toxicity criteria, further ensuring consistency in describing adverse outcomes. Complications were broadly categorized as minor or major, following the classification recommended by the Cardiovascular and Interventional Radiological Society of Europe (CIRSE). Across 296 MRI-FLA procedures, 38 complications were observed, representing an overall complication rate of 12.8%. Notably, only one major complication occurred (0.83%; 1 out of 296 procedures), as reported by Walser et al. [19], and no deaths associated with MRI-FLA were documented. The pooled prevalence of adverse effects (both minor and major) across studies was 14.26%, with a confidence interval ranging from 0.61% to 37.13% (*p* < 0.01), suggesting that MRI-FLA generally has a favorable safety profile with a manageable rate of complications.

Minor complications included bleeding, infection, pain, urinary retention, hematuria, urinary tract infection, recto-urethral fistula, transient paresthesia, hematospermia, retrograde ejaculation, and the need for medication to manage lower urinary tract symptoms or erectile dysfunction. Major complications were limited to severe urinary tract infections

### 3.7. Decrease in PSA

**Figure 5:**
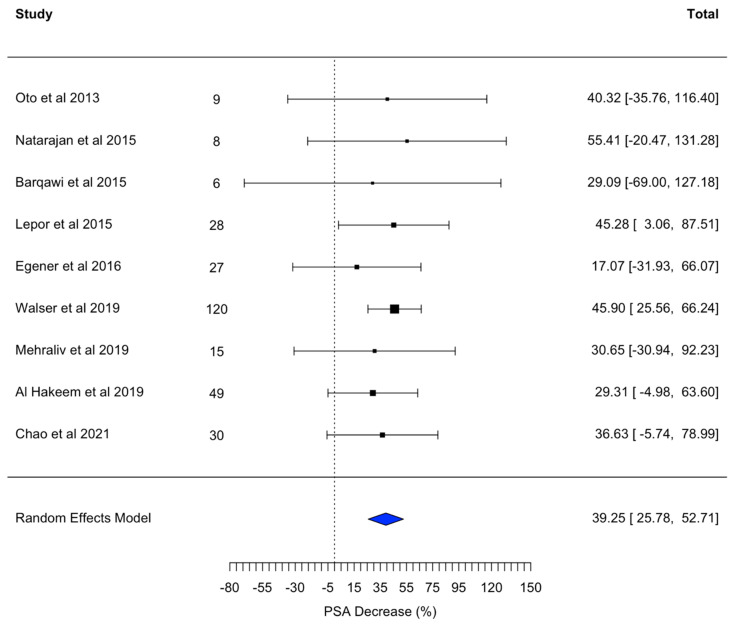
A random effects model for decrease in PSA after MRI-FLA [19,20,21,22,23,24,25,26,27].

Figure 5 illustrates a random effects model analyzing the decrease in prostate-specific antigen (PSA) levels following MRI-FLA. This model consolidates findings from multiple studies, adjusting for variations across them to provide an overall estimate of PSA reduction as a marker of treatment effectiveness. By utilizing the random effects approach, the analysis accounts for study heterogeneity, offering a robust view of the typical decrease in PSA levels post-MRI-FLA. This decrease in PSA serves as an indirect indicator of the ablation’s success in reducing tumor burden, demonstrating the potential of MRI-FLA to achieve measurable biochemical responses in treated patients.

A random effects model evaluating the pooled data from multiple studies demonstrated an overall decrease in prostate-specific antigen (PSA) levels of 39.25%, with a confidence interval ranging from 25.78% to 52.71%. This significant reduction in PSA suggests that MRI-FLA effectively reduces tumor burden in treated patients, as PSA levels often correlate with the presence and progression of prostate cancer.

### 3.8. Procedural Time

Six studies [19,20,21,23,24,27] reported data on the total procedure time for MRI-guided focal laser ablation (MRI-FLA), with a mean duration of 200 min and a range from 122 to 283 min. This variation in procedure time may reflect differences in patient characteristics, tumor complexity, and institutional protocols. The extended duration emphasizes the technical precision required for MRI-FLA, as well as the need for careful imaging, targeting, and real-time monitoring to ensure effective ablation while minimizing risk to surrounding tissues.

### 3.9. Follow-Up

The follow-up duration varied among the studies. The longest mean follow-up was 6 years (range: 4–7.2 years), reported by Chao et al. [26], and the shortest was 3 months, reported by Lepor et al. [25].

## 4. Discussion

All articles included in this study reported that the use of MRI-FLA to treat prostate tumors was safe and effective. In the context of FT for PCa, MRI is the most effective imaging modality; the images are of high resolution and clearly delineate prostate tumors [29,30]. Accurate lesion identification is critical for accurate and effective FT, which reduces the risk of incomplete ablation and preserves surrounding healthy tissue, optimizing patient outcomes. However, MRI is often expensive and is not always available. Thus, US, which is more accessible and significantly cheaper, is often the preferred imaging modality. US-guided and MRI–US fusion techniques are more commonly employed than MRI alone. The MRI–US fusion approach exploits the strengths of both modalities, enhancing tumor localization by MRI and real-time treatment guidance under US.

A key consideration during MRI- or US-guided FT is how the treatment margins are defined. Margins are additional tissues around a tumor that are also ablated to ensure the complete destruction of malignant cells. The use of appropriate margins is critical; margins that are too narrow may be associated with RD and recurrence, while excessively wide margins damage healthy tissue, thus affecting quality of life, including in terms of sexual function and urinary continence.

In our meta-analysis, the RD rate was 20.37% (12.56–29.28%; *p* = 0.03) after MRI-guided tumor ablation. Similarly, in 40 patients who underwent FLA under MRI-US guidance and MRI fusion, the RD rate was 20% at 36 months, and there was no recurrence [31]. Peretsman et al., in a systematic review of HIFU treatments for localized PCa, reported an RD rate of 19.8% (95% CI 12.4–28.3). Reintervention was required in 20–30% of patients [32]. Ghai et al. [33] reported that 91% of patients treated under MRI-US exhibited no clinically significant PCa in the ablation zone 2 years later, and 84% had no cancer in the entire gland. Bakavinicius et al., in a systematic review [34], reported that the second HIFU intervention rate was 4–19%.

MRI-FLA is a promising technique that precisely targets tumors with minimal damage to surrounding structures. MRI-FLA is associated with few major complications; the rate thereof is only 0.3%. In contrast, a recent review [35] of various FT modalities (cryotherapy, irreversible electroporation [IRE], HIFU, and photodynamic therapy [PTD]) reported erectile dysfunction rates of 3–50% and urinary incontinence rates of 0–34%. Barret et al. [36] found that the complication rate was 13% after HIFU, with 2% of the complications being grade three complications. Rakauskas et al. [37] found that urinary tract infections and acute urinary retention developed in up to 17% of cases; dysuria and hematuria were even more common. Urinary incontinence after FT is rare (0–5%). The meta-analysis of Walker et al. [38] found that whole-gland ablation was associated with a more significant decline in erectile function than was FT ablation, with only minimal improvement over 36 months. Zhang et al. [39] found that focal and extended laser ablation achieved comparable oncological outcomes in men with localized low- or intermediate-risk prostate cancer.

After MRI-FLA, the PSA level decreased by an average of 39.25% (range: 25.78–52.71%). Stabile et al. [40] reported a median PSA reduction of 73% following HIFU and noted that a reduction of <10% may indicate a need for retreatment. MRI-FLA procedures are relatively long (mean duration = 200 min [range of 122–283 min]). Ghai et al. [6] reported a median “magnet time” (from MRI to the recovery room) of 256 min, with a median ablation time of 125 min when transrectal MRI-guided focused US was employed. US-guided HIFU procedures tend to be shorter, typically <120 min [5,41,42]. The mean transurethral ultrasound ablation (TULSA) procedure time was reported as 161 min (range: 104–218 min) [43].

The visually directed high-intensity focused ultrasound (HIFU) approach shows promising parallels to MRI-guided focal laser ablation (MRI-FLA) in treating localized prostate cancer by enabling precise, focal therapy that minimizes damage to surrounding tissues. A recent meta-analysis reported by Peretsman et al. [44] highlights that although visually directed HIFU maintains acceptable cancer control rates, 16.7% of complications include erectile dysfunction and urinary incontinence, underscoring the importance of controlled, real-time adjustments during treatment. Comparing cancer control rates between focal therapies, HIFU showed a clinically significant positive biopsy rate of 19.8% on follow-up, with lower salvage treatment rates (8.6% for whole-gland). Some adverse effects after HIFU were reported on this metanalysis including erectile dysfunction (6.2%), urinary retention, 3.0%, urinary tract infection (1.9%), urinary incontinence (1.9%), and bowel injury (0.1%).

The major limitations of this meta-analysis include the heterogeneity of the included studies and the short follow-up. Furthermore, the same variables were not reported by all studies, so it was not easy to pool the results. While a Delphi consensus on focal therapies for prostate cancer has recommended a minimum follow-up duration of 5 years [45], none of the studies in this review included patient data extending over this period. Despite its promising results, MRI-FLA presents certain limitations, including extended procedure times, which may affect its practicality in routine clinical settings.

Our meta-analysis revealed that the included studies were at high risk of bias, which poses a significant limitation.

Our meta-analysis revealed a high level of inconsistency (I² > 50%) across all end-points. This heterogeneity is likely attributable to differences in population characteristics, including variations in patient selection criteria, tumor size, and treatment protocols. Future studies with more standardized patient cohorts and treatment protocols are needed to reduce variability and strengthen the evidence base

Consequently, long-term data on prostate cancer control outcomes for visually directed MRI-FLA remain limited, underscoring the need for further research to assess its sustained effectiveness.

## 5. Conclusions

MRI-FLA safely and effectively treats localized PCa; the prevalence of RD is comparable to that after other forms of FT. MRI-FLA is associated with minimal urinary incontinence and erectile dysfunction, and the procedure is safe. However, refinements would allow for more consistent and effective ablation, and the long-term outcomes require more attention. MRI-FLA is time-consuming and requires an MRI scanner, so it may not always be possible.

## Figures and Tables

**Table 1:** Studies, demographics and characteristics at inclusion [19,20,21,22,23,24,25,26,27].

Authors	Year	Patients	Mean Age (Year)	Mean Tumor Diameter (mm)	Mean PSA (ng/mL)	Mean Follow-Up	Cancer Free Survival	Residual Disease	Recurence Retreatment	Minor Complication	Major Complication	Mean PSA After Intervention	Mean Time Procedure (min)
Oto et al. [19]	2013	15	66	12	6.2	6	78	22	NA	26.7	0	3.7	190
Natarajan et al. [24]	2015	8	63	11.5	7.4	6	62	38	NA	23	0	3.3	292
Barqawi et al. [22]	2015	7	NA	NA	5.5	12	34	66	NA	14	0	3.9	NA
Lepor et al. [25]	2015	25	66	11	5.3	3	96	0	NA	28	0	2.9	NA
Eggener et al. [23]	2016	27	62	15	4.1	3	96	11	NA	28	0	3.4	197
Walser et al. [21]	2019	120	64	12	6.1	34	83	17	NA	12.5	1,6	3.3	122
Mehralivand et al. [26]	2019	15	66	12.25	6.2	36	53.3	47	NA	67	0	4.3	283
Al Hakeem et al. [27]	2019	49	63		5.8	18	79.6	20	NA	0	0	4.1	120
Chao et al. [20]	2021	36	66	11	5.5	71.5	83	17	40	NA	0	NA	NA

NA: Not Applicable.

## Data Availability

No new data were created or analyzed in this study.
